# Capsular Bag Performance of a Novel Hydrophobic Single-Piece Intraocular Lens

**DOI:** 10.1007/s40123-024-01075-x

**Published:** 2024-12-12

**Authors:** Klemens Waser, Klaus Straßmair, Leon Pomberger, Haidar Khalil, Peter Laubichler, Matthias Bolz, Nino Hirnschall

**Affiliations:** 1 Kepler University Clinic, Krankenhausstraße 9, 4020 Linz, Austria; 2 Johannes Kepler University, Linz, Austria; 3 Smile Eyes, Trier, Germany

**Keywords:** Capsular bag performance, Clareon IOL CNA0T0, Anterior chamber depth, Tilt, Decentration

## Abstract

**Introduction:**

We conducted an evaluation of capsular bag performance of the Clareon CNA0T0 intraocular lens (IOL), focusing on postoperative anterior chamber depth (ACD), IOL tilt, and IOL decentration.

**Methods:**

Inclusion criteria were bilateral age-related cataract and the ability to provide informed consent. Exclusion criteria were prior surgeries, combined surgeries, and conditions posing a risk for postoperative capsular bag instability. Preoperative and 8-week postoperative assessments included optical biometry and high-resolution anterior segment optical coherence tomography (OCT). Subjective refraction was conducted only at 8 weeks postoperative visit.

**Results:**

In the first analysis, 49 right eyes of 49 patients were included. Mean preoperative and postoperative ACD were 3.10 and 4.69 mm, respectively. Mean preoperative tilt was 4.77°, increasing to 5.06° postoperatively. Preoperative decentration was 0.16 mm, increasing to 0.26 mm postoperatively. Absolute refractive error (ARE) was + 0.31D, with 81% of eyes within ± 0.5D limits. In analysis II (98 eyes of 49 patients), both eyes showed a moderate correlation in IOL tilt (Pearson correlation coefficient: 0.27, *p* = 0.061) and a low correlation in IOL decentration (Pearson correlation coefficient: 0.02, *p* = 0.892) and ARE (Spearman: 0.15, *p* = 305) between right and left eyes of the same patient.

**Conclusions:**

The Clareon CNA0T0 IOL demonstrated high mechanical stability, with low postoperative tilt and decentration values, resulting in excellent refractive outcomes and visual acuity. These findings confirm the IOL’s high stability within the capsular bag and effectiveness in minimizing postoperative refractive error, requiring only minor A-constant adjustments for optimal cataract surgery outcomes.

**Trial Registration:**

NCT06595693.

## Key Summary Points

**Table:** 

Postoperative lens misalignment is a significant source of refractive error after cataract surgery, impacting patient satisfaction, particularly with premium intraocular lenses (IOL).
The study demonstrated that the Clareon CNA0T0 IOL exhibits high mechanical stability within the capsular bag, leading to low postoperative tilt and decentration values, which in turn resulted in excellent refractive outcomes and visual acuity.
Mean absolute refractive error (ARE) was + 0.31D, with 81% of eyes falling within ± 0.5 D. This indicates that the Clareon CNA0T0 IOL effectively minimizes postoperative refractive error, requiring only minor adjustments to the A-constant for optimal cataract surgery outcomes.
A moderate correlation in IOL tilt and a low correlation in IOL decentration were observed between the right and left eyes of the same patient.
Postoperative results showed excellent uncorrected (UCDVA) and corrected (DCVA) distance visual acuity (UCDVA logMAR 0.07 and DCVA logMAR - 0.06) at 8 weeks postoperatively.

## Introduction

In modern cataract surgery, an artificial intraocular lens (IOL) is implanted in the capsular bag. In recent years, cataract surgery has evolved from a curative procedure to an additional refractive one. Patient satisfaction after uneventful cataract surgery strongly depends on the postoperative refraction. This effect is increased in the case of so-called premium IOLs [1–3].

According to current literature, the most significant source of postoperative refractive error is a postoperative misalignment of the lens [4]. Misalignment of the lens within the capsular bag, depending on the dimension of misalignment and the lens design, leads to reduced visual and optical performance of the lens. Axial misalignment results in defocus and lower-order aberrations. Transverse misalignment (decentration) and tilt, beyond a physiological threshold, predominantly induces higher-order aberrations, significantly hindering the optimal performance of aspheric, toric, and premium lenses [5].

The stability of an IOL within the capsular bag is significantly influenced by the lens design (1-piece vs. 3-piece IOLs), the haptic design (plate vs. C-loop haptic), and the angulation or off-set between the optic and the haptic [6–8].

This study aimed to evaluate the capsular bag performance of a new hydrophobic single-piece IOL (Clareon CNA0T0) regarding postoperative anterior chamber depth (ACD), IOL tilt, and IOL decentration.

## Methods

The present study was a prospective, single-center investigation. It included patients with bilateral age-related cataracts who were scheduled for cataract surgery on both eyes at Kepler University Hospital Linz. Inclusion criteria required capacity for informed consent and a signed patient information sheet after sufficient consideration time. Exclusion criteria were planned combined surgeries (glaucoma surgery, vitreoretinal surgery, or corneal surgery), best-corrected visual acuity (Snellen equivalent) of less than 0.1 preoperatively, or pregnancy. Further exclusion criteria were conditions and situations associated with increased risk of postoperative capsular bag instability, such as pseudoexfoliation syndrome (PEX), prior ophthalmologic trauma, or phacodonesis, as well as previous surgeries such as pars plana vitrectomies. A valid ethics vote of the regional ethics committee was obtained (EK Nr. 1044/2023) and the study adheres to the Declaration of Helsinki.

Preoperatively, and 8 weeks postoperatively, a comprehensive set of assessments was conducted. This included optical biometry (IOLMaster 700, Carl Zeiss Meditec AG, Germany), high-resolution anterior segment optical coherence tomography (OCT) (CASIA II, Tomey, Japan and MS-39, CSO, Italy), and Scheimpflug imaging (Pentacam HR, Oculus, Germany). Additionally, autorefraction was performed at each time point. Subjective refraction and visual acuity assessment was conducted 8 weeks postoperatively. Visual acuity measurements included uncorrected distance visual acuity (UCDVA) and intermediate distance (66 cm) visual acuity (UCIVA), as well as best-corrected distance visual acuity (DCVA), which was also measured at an intermediate distance (66 cm) (DCIVA). Visual acuity assessment was conducted monocularly and binocularly. IOL tilt and IOL decentration were measured using the Casia II. It should be noted that the Casia II uses a line perpendicular to the corneal vertex as the reference axis for determining lens tilt and decentration.

Cataract surgeries were performed by four experienced surgeons under topical anesthesia. Preoperative instillation regimen consisted of tropicamide, phenylephrine, and cyclopentolate. Surgeries were exclusively conducted from the superior approach, with a corresponding placement of the clear corneal incision (2.5 mm) at the 12 o'clock position. Paracenteses were done accordingly at the 2–3 o'clock and 9–10 o'clock positions. Utilizing an ophthalmic viscosurgical device (ProVisc, Alcon, USA), circular continuous capsulorhexis was done with the cystotome. After hydrodissection, phacoemulsification was performed followed by irrigation/aspiration for cortex removal. All patients received the same type of IOL (Clareon CNA0T0, Alcon, USA), into the capsular bag using AutonoME^®^ automated delivery system. Afterwards, the viscoelastic substance was carefully removed from the anterior chamber and behind the lens.

The new Clareon CNA0T0 IOL manufactured by Alcon is a foldable, monofocal, aspheric (– 0.2 µm), hydrophobic acrylic lens with a planar C-loop haptic, 6-mm optic, and a total length of 13 mm. The lens material is derived from the hydrophobic AcrySof polymer, employing milling techniques for edge profiling and quality enhancement, along with a modification in equilibrium water content (1.5% at 35 °C) and a refractive index of 1.55. Furthermore, the material includes an ultraviolet blocker and a chromophore that filters blue light [9, 10]. Numerous studies have been published evaluating the safety of this lens in conjunction with the automated delivery system AutonoME [9, 11].

The IOL constants used in this work were derived from IOLCon website (“https://iolcon.org”).

### Analysis

Two different sub-analyses were conducted. In the first analysis, only one eye per patient was included, specifically the right eye. With this dataset, descriptive statistics were performed and the effects of misalignment of the IOL in different dimensions on postoperative refractive error were examined.

In a second analysis, both eyes of each patient were included. This analysis aimed to elucidate the associations of IOL misalignment between the right (OD) and left eye (OS), as well as to examine the postoperative visual acuity of individual patients. For parametric data, Pearson's correlation was employed to assess potential correlations, while for non-parametric data, Spearman's rank test was used.

## Results

In the present study, a total of 104 eyes from 52 patients were included. A total of three patients were excluded from the analysis, resulting in data from 98 eyes of 49 patients being evaluated. Two of the patients did not attend the postoperative follow-up and were thus not included in the analysis. The third patient did not disclose a severe eye trauma during recruitment and subsequently developed intraoperative zonulolysis.

Among the participants, 20 were male and 29 were female. The mean age was 71.80 (± 7.87) years. All 49 patients underwent uneventful implantation of the Clareon CNA0T0 IOL into the capsular bag of both eyes. Biometric and IOL alignment data are summarized in Table 1.

**Table 1:** Biometric data (IOL Master 700^a^) and intraocular lens (IOL) alignment data (Casia II^b^)

	Right eyes	Left eyes
Axial eye length (mm)(mm)^a^	23.14 (± 0.93)	23.06 (± 0.95)
K mean preoperative (preop) (D)^a^	44.19 (± 1.54)	44.26 (± 1.55)
K mean postoperative (postop) (D)^a^	44.20 (± 1.56)	44.27 (± 1.57)
Lens thickness preop (mm)^a^	4.62 (± 0.41)	4.67 (± 0.39)
Anterior chamber depth (ACD) preop (mm)^a^	3.10 (± 0.38)	3.07 (± 0.38)
ACD postop (mm)^a^	4.69 (± 0.34)	4.66 (± 0.36)
Lens tilt amount preop (°)^b^	4.77 (± 1.51)	5.20 (± 1.38)
Intraocular lens (IOL) tilt amount postop (°)^b^	5.06 (± 1.14)	5.49 (± 1.16)
Tilt amount difference (°) (postop-preop)	0.29 (± 1.32)	0.29 (± 1.21)
IOL decentration preop (mm)^b^	0.16 (± 0.08)	0.15 (± 0.09)
IOL decentration postop (mm)	0.26 (± 0.13)	0.26 (± 0.15)
IOL power [D]	21.82 (± 2.33)	22.02 (2.61)
Decentration amount difference (mm) (postop-preop)	0.094 (± 0.146)	0.11 (± 0.16)
Mean manifest spherical equivalent (= SE) postop (D)	0.20 (± 0.44)	0.24 (± 0.43)
Mean relative refractive error (manifest SE postop- estimated SE postop by Kane formula) (D)	0.03 (± 0.39)	0.07 (± 0.40)
Mean absolute refractive error (Kane formula) (D)	0.31 (± 0.23)	0.31 (± 0.26)

### Analysis I: Right Eyes Only

This analysis included 49 eyes from 49 patients. Mean axial eye length (AL) was 23.14 mm (± 0.93). Preoperative ACD and postoperative ACD was 3.10 mm (± 0.38) and 4.69 mm (± 0.34), respectively.

**Fig. 1:**
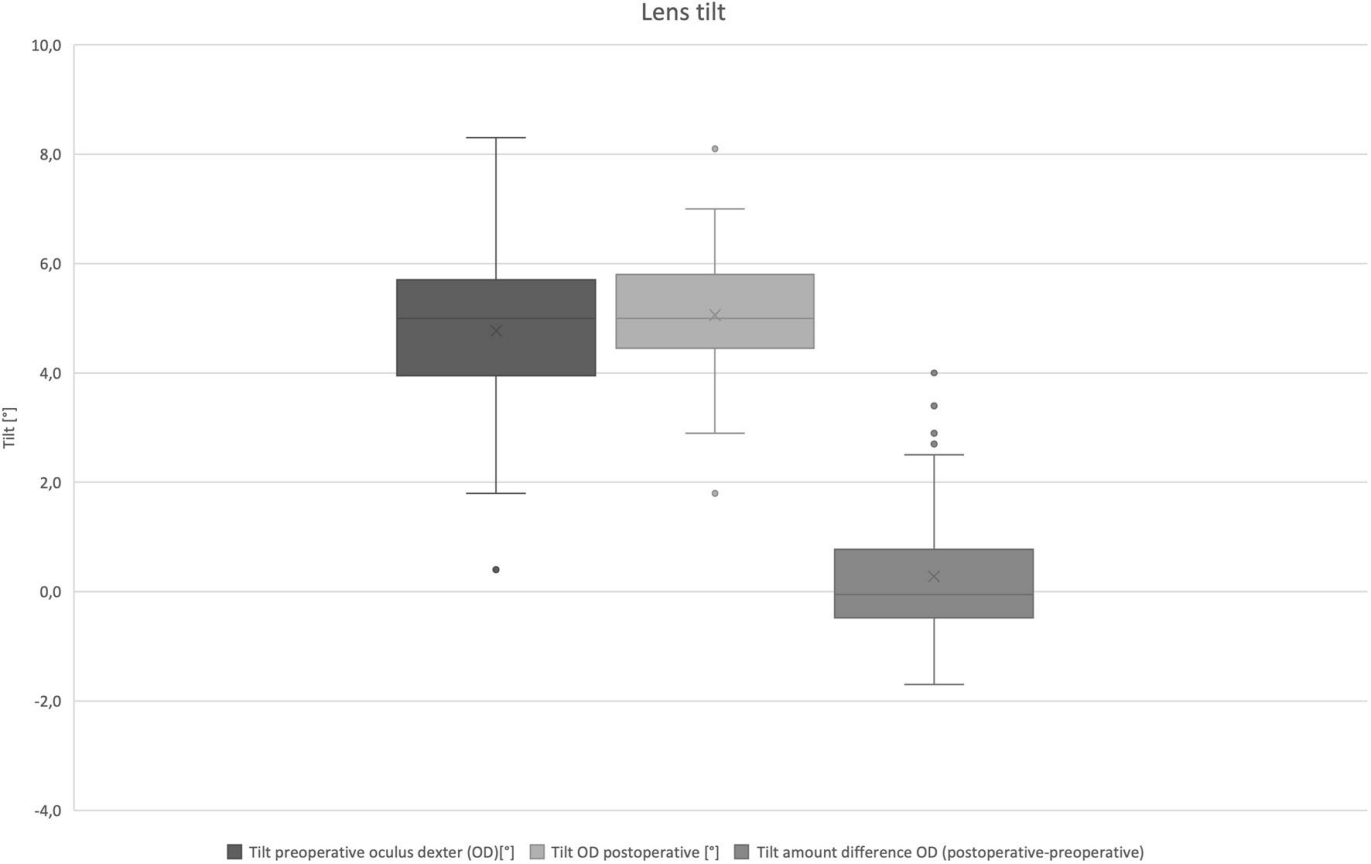
Distribution of lens tilt amount (°) of right eyes (OD) only: tilt difference amount was calculated postoperative tilt minus preoperative tilt. A positive prefix represents an increase of tilt postoperatively

The mean preoperative tilt amount was 4.77° (± 1.51). Distribution of lens tilt is presented in Fig. 1. Preoperative orientation of the crystalline lens tilt was superonasal in one case, superotemporal in six cases, and inferotemporal in 42 cases. An orientation of the lens tilt towards temporal inferior means that the optical axis of the lens is tilted towards temporal inferior compared to the reference axis. In other words, the temporal inferior edge of the optic is shifted posteriorly, while the opposite superonasal part of the optic is shifted anteriorly.

The mean postoperative tilt was 5.06° (± 1.14). Postoperative tilt values ranged from 1.8° to 8.1°. Postoperative orientation of the IOL tilt was superotemporal in seven cases, and inferotemporal in 42 cases.

**Fig. 2:**
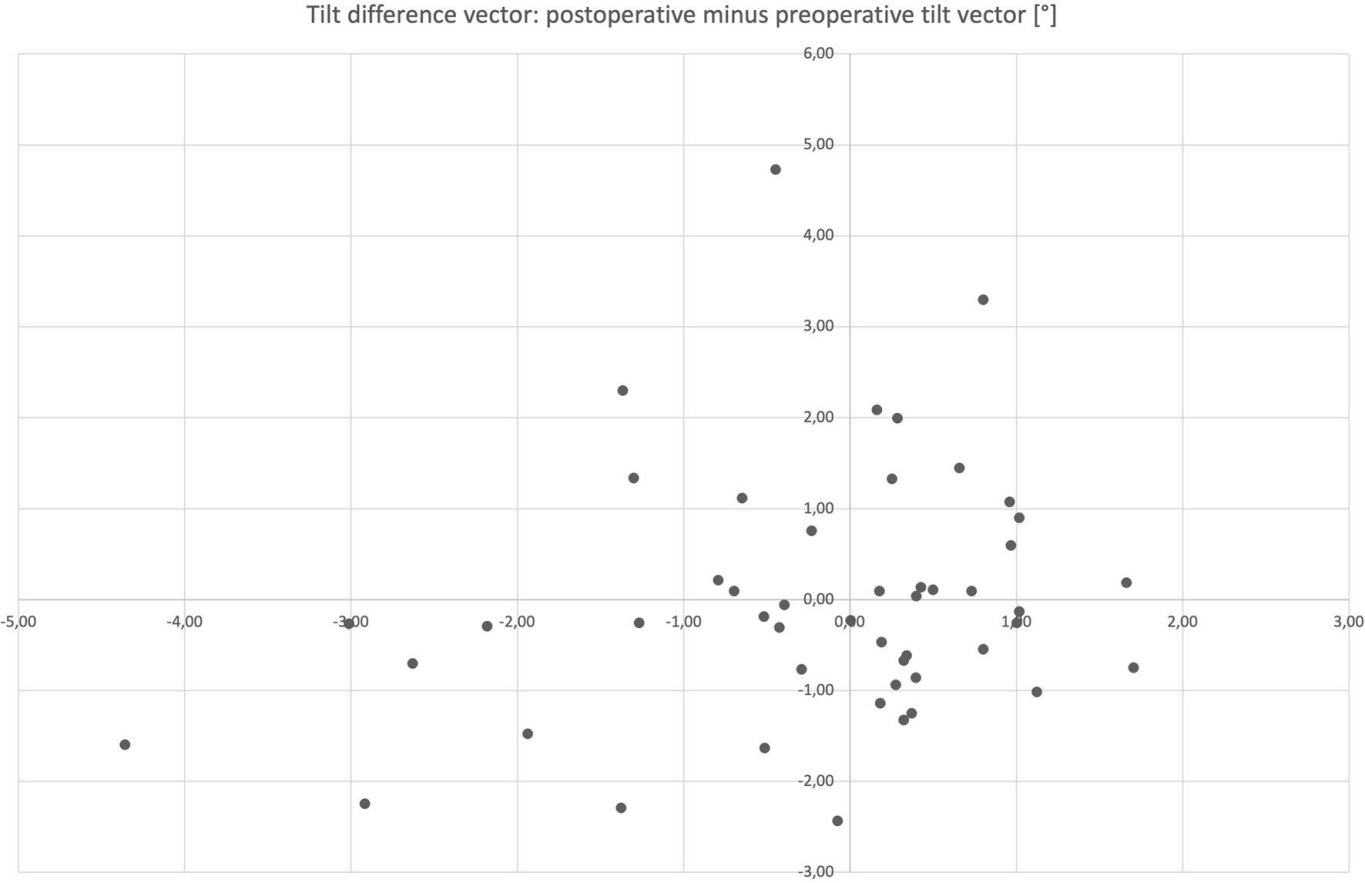
Tilt difference vector (°) of right eyes only: postoperative minus preoperative tilt. A positive prefix represents an increase of tilt postoperatively

**Fig. 3:**
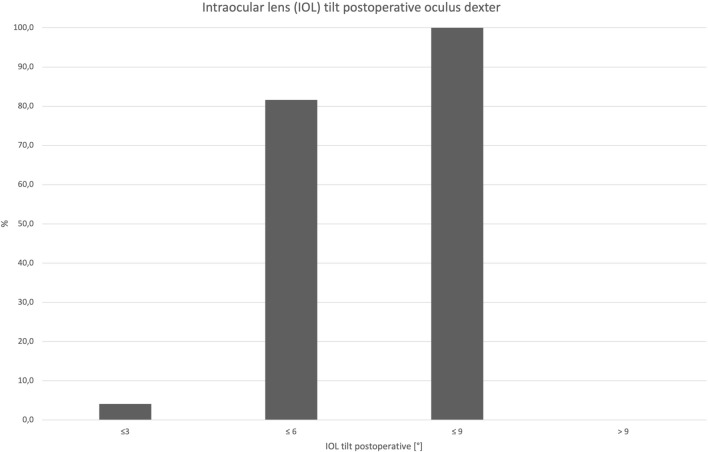
Cumulative percentage of postoperative IOL tilt (°) of right eyes only

The mean difference of postoperative minus preoperative tilt amount was 0.29° (± 1.32). Tilt difference vector is shown in Fig. 2. Postoperative cumulative percentage of tilt amount is shown in Fig. 3.

**Fig. 4:**
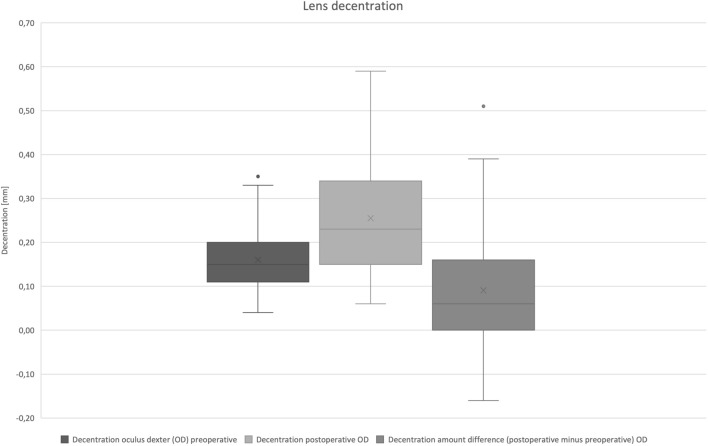
Distribution of lens decentration (mm) of right eyes only: decentration amount difference was calculated postoperative decentration minus preoperative decentration. A positive prefix represents an increase of decentration postoperatively

Preoperative lens decentration was 0.16 mm (± 0.08). Distribution of lens decentration is presented in Fig. 4. Orientation of the preoperative decentration was superonasal in nine cases, superotemporal in 22 cases, inferotemporal in 11 cases, and inferonasal in seven cases. Postoperative IOL decentration was 0.26 mm (± 0.13). Orientation of postoperative decentration was superonasal in 11 cases, superotemporal in 20 cases, inferotemporal in 12 cases, and inferonasal in six cases.

**Fig. 5:**
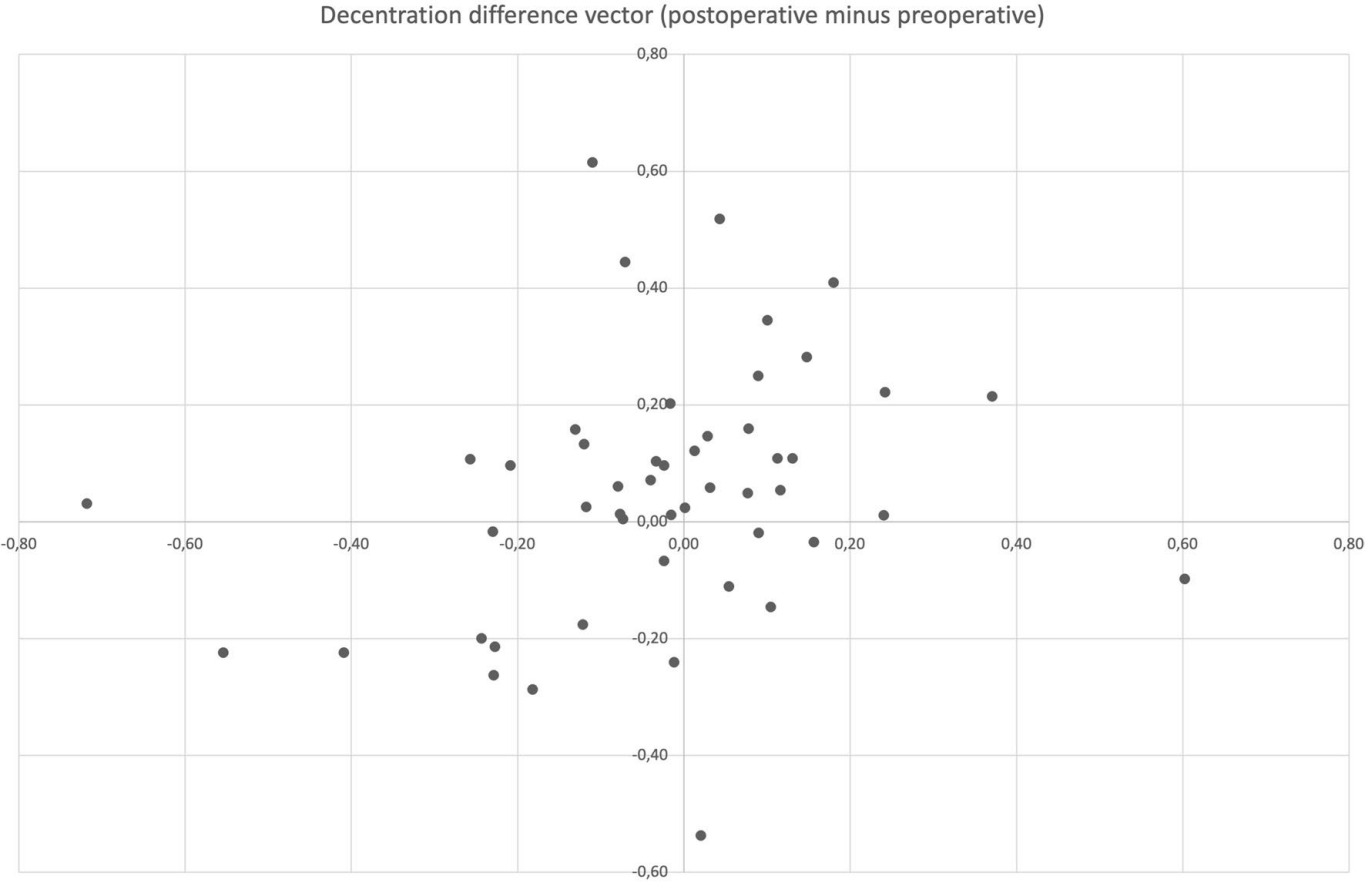
Decentration difference vector (mm) of right eyes only: postoperative minus preoperative decentration. A positive prefix represents an increase of decentration postoperatively

**Fig. 6:**
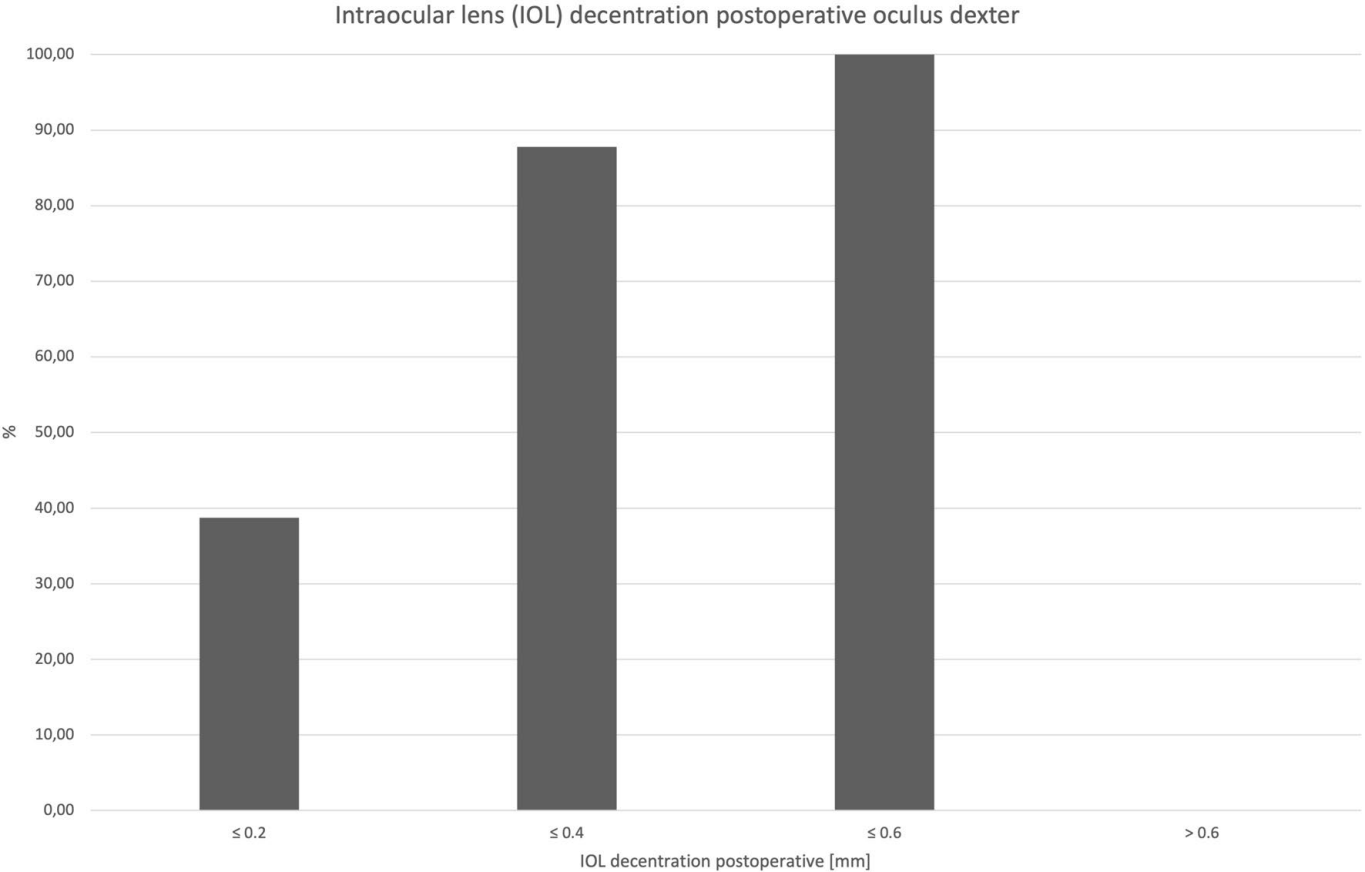
Cumulative percentage of tilt (mm) of right eyes only

The difference of IOL decentration was 0.09 mm (± 0.146). Decentration difference vector is shown in Fig. 5. Postoperative cumulative percentage of decentration is shown in Fig. 6.

**Fig. 7:**
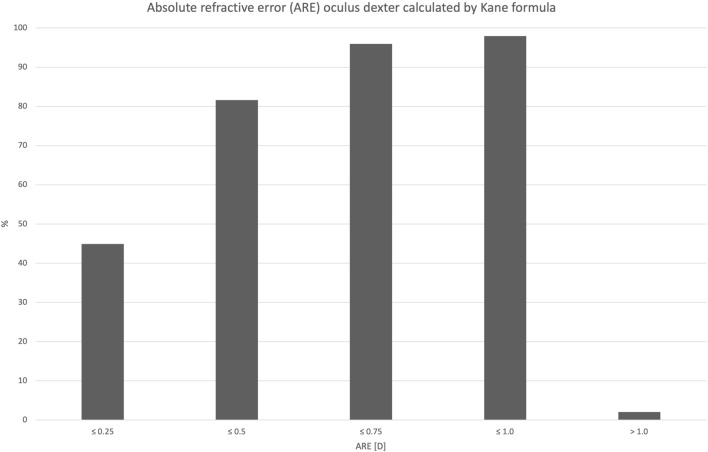
Cumulative percentage of absolute refractive error (ARE) using Kane formula: ARE calculation: manifest postoperative spherical equivalent minus predicted refractive error calculated. To prevent myopic and hyperopic errors from canceling each other out, the absolute value was derived

**Fig. 8:**
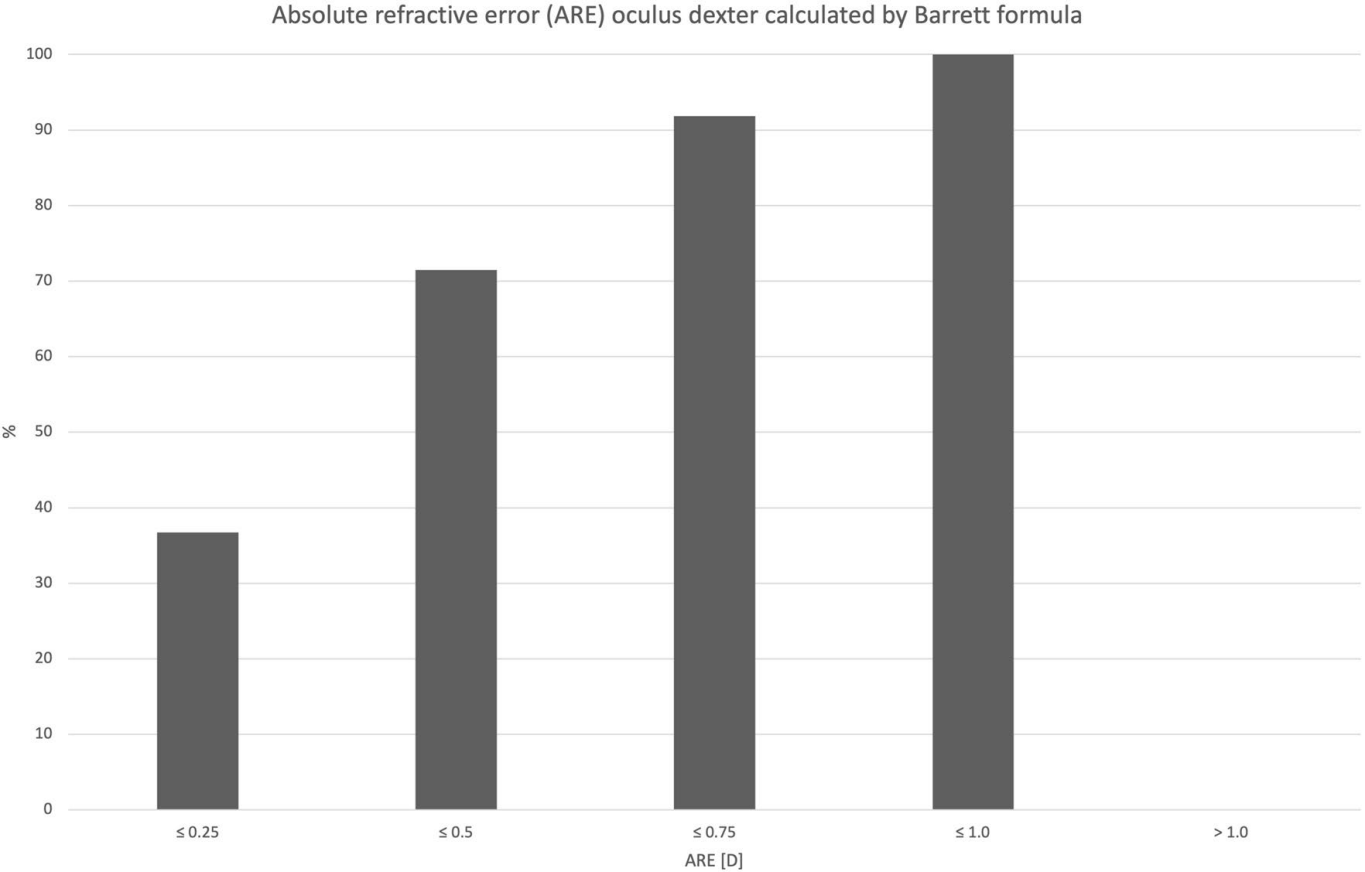
Cumulative percentage of ARE using Barrett formula of right eyes only

Mean used IOL power was 21.82 D (± 2.33) with a range from 18.0 to 27.5 D. Preoperatively calculated target refraction for implanted IOLs using Kane calculation formula was + 0.17 D (± 0.18). Mean manifest spherical equivalent (SE) at the subjective refraction 8 weeks postoperatively was + 0.20 D (± 0.44). This results in a mean relative refractive error (RRE) of 0.03D (± 0.39) and an absolute refractive error (ARE) of + 0.31 D (± 0.23). Cumulative percentage of ARE is shown in Fig. 7 using Kane formula. Figure 8 shows the cumulative percentage of ARE calculated by using Barrett formula. Median ARE was + 0.27 D. The Spearman rank correlation coefficient between ARE and AL is – 0.107. At the subjective refraction 8 weeks postoperatively mean UCDVA was logMAR 0.07 (± 0.13) and DCVA was logMAR – 0.06 (± 0.08); 81% of all right eyes showed an ARE within ± 0.5 D limits.

### Analysis II: Visual Acuity and Intraindividual Analysis: Right Eyes (OD) vs. Left Eyes (OS)

In a second analysis, the associations of misalignment of the lens between the right and left eye within an individual were investigated. This analysis included 98 eyes from 49 patients.

Only a slight difference in postoperative SE was found between right and left eyes. Spearman rank correlation test between ARE of right and left eyes was 0.15 (*p* = 305). Pearson correlation coefficient between right and left eyes of the difference in IOL tilt and the difference of IOL decentration was 0.27 (*t* test: *p* = 0.061) and 0.02 (*t* test: *p* = 0.892), respectively (Table 1).

There was no difference in mean DCVA between right and left eyes and a slight improvement of visual acuity in binocular testing. Only a slight difference was found in UCDVA and DCIVA between right and left eyes (Table 2).

**Table 2:** Visual acuity

	Right eyes	Left eyes	Both eyes
Spherical equivalent postop (D)	0.20 (± 0.44)	0.24 (± 0.43)	0.22 (± 0.38)
Best corrected distance visual acuity [logMAR]	− 0.06 (± 0.09)	− 0.06 (± 0.09)	− 0.09 (± 0.08)
Uncorrected distance visual acuity [logMAR]	0.08 (± 0.13)	0.10 (± 0.13)	0.01 (± 0.11)
Uncorrected intermediate visual acuity [logMAR] (66 cm)	0.32 (± 0.16)	0.34 (± 0.15)	0.23 (± 0.15)
Distance corrected intermediate visual acuity [logMAR] (66 cm)	0.30 (± 0.14)	0.28 (± 0.14)	0.18 (± 0.13)

As previously mentioned, there was an intraoperative adverse event during the study involving zonulolysis in a patient who had not disclosed a severe eye trauma during recruitment. This patient was excluded from the study. Another patient experienced mild Descemet membrane detachment at the position of the incision, which was managed successfully with re-bubbling in the postoperative course. This patient remained in the study. These adverse events were reported to Alcon in accordance with the customer complaint adverse event reporting requirements.

## Discussion

In the present study, capsular bag stability of the new Clareon CAN0T0 IOL using high-resolution OCT in real-life patients with cataract from daily routine practice was investigated. This new IOL demonstrated high mechanical stability, low absolute refractive error, as well as excellent visual acuity outcomes.

Negishi et al. described a high axial stability of the Clareon CNA0T0 in their study, attributed to the stable postoperative anterior chamber depth over the postoperative course, which resulted in very good visual acuity outcomes [11]. The anterior chamber depth in the Negishi study increased on postoperative day one, then decreased until postoperative week 1, and remained stable for the rest of the postoperative period. Because the anterior chamber depth is constant after 1 week, it can be assumed that an assessment of the long-term lens position at 8 weeks postoperatively is an accurate time point. In our study, lens tilt and transverse stability (decentration) were measured and correlated not only with visual acuity but also with the absolute refractive error, as it is a better indicator for capsular bag performance as visual acuity alone (for a detailed explanation, see below in the text). However, the anterior chamber depth results reported by Negishi et al. were confirmed in our study and, along with a low mean absolute refractive error, indicate a high axial stability of the Clareon CNA0T0.

Lane et al. investigated the mechanical stability of five different single-piece monofocal lenses, including the Clareon CNA0T0 IOL and the AcrySof SN60WF, a predecessor of the Clareon, in an in vitro analysis [6].

Both IOLs feature planar haptics, in contrast to the other three lenses studied, which have either step vault haptics or angulated haptic designs. In this study, the axial stability, tilt, and decentration of the various lenses were measured under standardized pressure diameters, and the resulting calculated refractive error was evaluated. The research group concluded that the CNA0T0 IOL and the AcrySof SN60WF, due to their planar haptic design with a flexible hinge, showed a significantly reduced axial displacement compared to the other lenses. Consequently, the theoretically calculated refractive error resulting from axial IOL shift was lower with the Clareon CNA0T0 IOL and AcrySof SN60WF than with the other lenses. The experiments demonstrated no significant difference in tilt and decentration among all lenses examined.

Interestingly, no difference in stability between the Clareon IOL and AcrySof SN60WF IOL was observed. The significant improvement in the Clareon IOL over AcrySof lies in the use of a material designed to reduce glistening. A previous study by Oshika et al. demonstrated that while Clareon is based on the AcrySof platform, it incorporates a hydrophilic copolymer, 2-HEMA, instead of the phenylethyl methacrylate used in AcrySof IOLs [12]. The higher water content of the Clareon IOL compared to AcrySof does not appear to influence its flexibility or behavior within the capsular bag.

However, the study of Lane et al. was conducted in vitro. The clinical results of our study confirm the assumption of high mechanical stability of the CNAT0T0 lens and demonstrate excellent refractive outcomes in real-life patients with cataract.

A multicenter study with a large dataset of 215 patients on the effectiveness and safety of the Clareon CNA0T0 was conducted by Nuijts [13]. As a long-term post-market analysis, tilt and decentration over a 3-year postoperative period were evaluated as secondary outcome parameters by measuring tilt in 1-degree increments and decentration in 0.5 mm increments using a light source and Purkinje reflexes under miosis. This method, published by Guyton, can be useful as a rough screening tool to detect high deviations in lens tilt or decentration [14]. However, their results only addressed the increase in tilt and decentration postoperatively, with absolute values not provided. According to their findings, two eyes exhibited a change in tilt and two eyes exhibited a change in decentration. Although these results are not directly comparable with our findings, the small number of eyes with a notable increase in tilt and decentration postoperatively suggests stable conditions. On the same principle of interpretation of the ratio of Purkinje reflections of the cornea to those of the lens, the Purkinje meter operates. The numerical analysis of tilt and decentration is performed objectively without examiner dependency. The primary disadvantage of the Purkinje meter is its limited availability outside of scientific institutions. Nevertheless, in this study, the mechanical stability of the Clareon IOL in the capsular bag was assessed by measuring the ACD, tilt, and decentration using the Casia II (Tomey, Japan), a second-generation high-resolution anterior segment OCT. According to recent studies, this technology, which is commonly available in clinical settings, enables precise and highly reproducible measurement of tilt and decentration of both the crystalline lens and IOL following cataract surgery [15, 16].

The influence of tilt and decentration on the optical performance of a lens is significantly dependent on the lens design. Aspheric lenses are more sensitive to tilt and decentration than spherical lenses, and multifocal lenses (mIOL) are more sensitive than lenses with an enhanced depth of focus (EDOF), which in turn are more sensitive than monofocal lenses [17]. According to current literature, a lens tilt greater than 7° and a decentration of the lens greater than 0.4 mm is assumed to be clinically significant, as it leads to deterioration in visual performance due to the induction astigmatism, and higher-order aberrations [18, 19]. In general, IOL tilt and decentration results were low. In our study collective of Analysis I,100% (49/49) exhibited a tilt value of ≤ 7°, and 87.76% (43/49) showed a decentration of ≤ 0.4 mm. These low values of tilt and transversal misalignment demonstrate the high stability of the Clareon lens within the capsular bag.

Visual outcomes and postoperative SE in subjective refraction 8 weeks after implantation of the Clareon CNA0T0 were excellent. Mean UCDVA and DCVA were exactly in line with the 3-month postoperative data from Negishi et al. and the 12-month data from Kim et al. Postoperative manifest SE from Negishi et al. was slightly myopic, whereas the manifest SE from our study was slightly hyperopic.

To allow for a better assessment of capsular bag performance, our study additionally calculated the relative refractive error (RRE) by subtracting the manifest SE from the predicted postoperative error, calculated by using the Kane formula and Barrett formula. To prevent myopic and hyperopic errors from canceling each other out, the absolute refractive error (ARE) was determined from this, shown in Fig. 7 by using Kane formula and Fig. 8 by using Barrett formula. As ARE represents the actual discrepancy between predicted and manifest errors, it is an accurate parameter for capsular bag stability interpretation. ARE is small in our cohort, leading to the conclusion that only minimal adjustment of the A-constant would be needed in our cohort.

Observations show that the right and left eyes of the same individual exhibit only a moderate correlation regarding IOL tilt difference, aligning with the results of a recent study by our research group on the predictability of lens tilt [20]. Interestingly, decentration difference and ARE also appear to have only a low correlation between the right and left eyes of the same patient.

A limitation of our study is the relatively small patient cohort. As these are real-life data, patient groups with short and long eyes are particularly underrepresented. Further studies are certainly needed to investigate the postoperative behavior of the lens in the capsular bag as refractive surprises are most common in these groups.

In this study, a high mechanical stability of the Clareon CNA0T0 IOL within the lens capsular bag was demonstrated, resulting in a low ARE. Only a minor adjustment of the A-constant was required in the present cohort.

## Conclusions

The Clareon CNA0T0 intraocular lens (IOL) demonstrated high mechanical stability within the capsular bag, evidenced by low postoperative tilt and decentration, resulting in excellent refractive outcomes and visual acuity. The study confirms that the Clareon CNA0T0 IOL is effective in minimizing postoperative refractive errors, requiring only minimal adjustments to the A-constant for optimal cataract surgery results. These findings underscore the IOL's reliability and support its use in clinical practice.

However, the study has some limitations. The relatively small patient cohort, especially the underrepresentation of patients with particularly short or long eyes, may limit the generalizability of the results. Additionally, further research with larger and more diverse patient populations is necessary to fully understand the postoperative behavior of the Clareon CNA0T0 IOL, particularly in cases where refractive surprises are more likely. Despite these limitations, the study provides valuable insights into the stability and effectiveness of the Clareon CNA0T0 IOL in routine cataract surgery.
